# Clinical Implications of Neuroplasticity – The Role of Rehabilitation in Multiple Sclerosis

**DOI:** 10.3389/fneur.2015.00036

**Published:** 2015-03-03

**Authors:** Peter Flachenecker

**Affiliations:** ^1^Neurological Rehabilitation Center Quellenhof, Bad Wildbad, Baden-Württemberg, Germany

**Keywords:** multiple sclerosis, rehabilitation, neuronal plasticity, physiotherapy, neuropsychological therapy, exercise therapy, functional imaging studies

Multiple sclerosis (MS) is a chronic autoimmune disease of the central nervous system (CNS) that preferably affects young adults and causes a multitude of symptoms including visual disturbances, spasticity, weakness, impairment of walking, coordination difficulties, tremor/ataxia, sensory problems, and bladder disturbances. In addition, “invisible” symptoms such as fatigue, depression, and cognitive dysfunction are also common and may even be present early in the course of the disease (1). These symptoms often cause huge disability and have an impact on family, social, and work activities. Despite the advances of pharmacological treatment, particularly by disease-modifying therapies, the majority of MS patients accumulate new lesions and disabilities along the disease course and thus, there is a continuing need for comprehensive, multidisciplinary treatment, which constitutes the basic concept of rehabilitation (2).

Rehabilitation is defined as a “problem-solving educational process aimed at reducing disability and handicap experienced by someone as a result of disease or injury” (3). The primary goal is to reduce the limitations of activity and participation in order to achieve the highest possible level of independence and to increase and maintain quality of life of MS patients (4). With respect to the large variety of symptoms, a multidisciplinary approach is required for MS rehabilitation that includes physiotherapy, occupational therapy, cognitive rehabilitation, psychological therapy, speech therapy, measures for improving fatigue, and coping programs (2, 5). These measures facilitate the reorganizing mechanisms within the CNS and therefore, rehabilitation may be regarded as “applied neuroplasticity.” This article gives an overview of the most recent scientific evidence and measures of MS rehabilitation, and the relationship between neuroplasticity and functional improvement in MS.

## Multidisciplinary Rehabilitation in MS

There is a large interest in scientifically sound studies dealing with the effectiveness of neurorehabilitation. During the last decades, a growing body of research has been performed, mainly in stroke patients, but also in MS. A recent update of a Cochrane review identified 10 randomized controlled trials dealing with multidisciplinary rehabilitation in MS (6). Although data are limited, the available evidence suggests that inpatient rehabilitation may have short-term effects on activity and participation, but not on impairment. Furthermore, there was “moderate evidence” to support inpatient or outpatient rehabilitation programs to improve disability, bladder dysfunction, and participation that may last up to 12 months. Since these effects diminish with time (7), repetition of multidisciplinary rehabilitation seems necessary, preferably on an annual base.

## Physiotherapy and Exercise Therapy

Physiotherapy is one of the basic methods of MS rehabilitation and aims at improving motor function, stability of gait, and walking capabilities. Moreover, endurance and physical fitness may also be strengthened and thus, fatigue may be ameliorated. There are many techniques and methodologies based on neurophysiological concepts (i.e., Bobath, Vojta, Brunkow, and proprioceptive neuromuscular stimulation) as well as newer approaches such as equipment-supported training, treadmill exercises, robot-assisted gait training, and constraint-induced movement therapy (CIMT) (2). Neither of these techniques has shown superiority about another which means that the appropriate method should be chosen according to the capabilities and disabilities of the individual patient, but also to the knowledge and resources of the rehabilitation team. Physiotherapy may also improve breathing dysfunction and bladder disturbances by using training programs specifically directed toward respiratory muscle and pelvic floor function, respectively (2, 8).

In numerous studies, the beneficial effects of exercise therapy for persons with MS have been shown. Despite methodological problems (small sample sizes, heterogeneous groups of patients, different interventions), there is good evidence that exercise has positive effects on balance (9), mobility (10), muscle weakness (11–13), depression (14), and fatigue (15). Therefore, persons with MS should be encouraged to participate regularly in endurance and/or resistance training of low to moderate intensity. These interventions are well tolerated and not associated with side effects (16, 17), but could positively influence both, the limitations caused by the disease itself and the additionally deconditioning effects of an inactive lifestyle.

## Cognitive Dysfunction and Fatigue

Cognitive dysfunction often accompanies the symptomatology of MS and is not necessarily associated with motor disability. It may occur early in the disease course and significantly affects employment, social life, and the activities of daily living (18). The most commonly affected areas are information processing speed, attention, memory, visuo-constructive performance, and executive functions (19). It is of utmost importance to recognize these problems as early as possible by appropriate neuropsychological tests, and to tailor the rehabilitation measures specifically toward the cognitive deficit. Since drug treatment is disappointing [the promising effects of the anti-cholinesterase agent donepezil could not be reproduced in a large randomized controlled trial (20)], treatment consists of neuropsychological training, provision of aids, and supportive psychotherapy [RIMS (21)]. Albeit with limited evidence, a systematic review indicated that cognitive training can improve memory span, working memory, and immediate visual memory (22). Moreover, benefits were found for specific trainings of attention, executive functions, learning performance, and memory (23, 24).

Fatigue is one of the most common and debilitating symptoms in MS and clearly different from normal tiredness. Patients suffer from feelings of lassitude and abnormal tiredness that may increase during the day as well as lack of energy and motivation, which all may impact activities of daily living and work ability [RIMS (21)]. The pathogenesis is still unknown and may involve different mechanisms such as lesions of cortical and/or subcortical motor pathways with involvement of motor cortex and basal ganglia, decreased energy metabolism in the frontal cortex, autonomic dysfunction, endocrine disturbances, and dysregulation of the hypothalamus–pituitary–adrenal axis [(25), RIMS (21)]. These “primary” fatigue needs to be differentiated from secondary mechanisms such as sleep disorders, anemia, and thyroid dysfunction, but also from depression and cognitive deficits. The subjective dimension of fatigue may be evaluated with standardized questionnaires, and attention tests of alertness may be an objective assessment method (26). Drug treatment is often not efficient. Therefore, management of fatigue consists of non-pharmacological measures such as counseling of patients and caregivers, structuring the day with regular breaks, energy management programs, cooling, specific neuropsychological training (attention), and exercise therapy (26).

## Neurorehabilitation as “Applied Neuroplasticity”

Within the last years, our knowledge about the basic mechanisms that may be responsible for the restoration of neurological disabilities is rapidly increasing. It is now generally accepted that even the mature brain can undergo plastic changes (27). Although the majority of studies are dedicated to the dynamic reorganization of the motor system after an acute event, i.e., stroke (28), these neuroplastic changes may also occur in a chronic disease as it is MS. For instance, brain activation was exaggerated in MS patients with normal motor function compared to healthy controls by using a finger tapping paradigm (29). The brain activation pattern changes with both, increasing diffuse brain injury (assessed by relative *N*-acetylaspartate concentration, a marker of axonal integrity) and increasing hand disability, and was present during active as well as passive finger movements reflecting true brain reorganization (30). The same applies for cognitive function: while MS patients in the early stages of MS performed similarly to healthy controls on clinical outcomes and the visual analog of the Paced Auditory Serial Addition Test (PASAT), brain activation was increased in the patient group indicating that compensatory adaptive mechanisms (i.e., neuronal plasticity) may be present very early in the course of MS (31).

Zeller et al. tried to elucidate the basic mechanisms underlying neuronal plasticity in MS. For this purpose, rapid-onset central motor plasticity was assessed in 22 patients with moderately severe, stable MS and compared to healthy controls using paired associative stimulation (PAS), a protocol that models long-term synaptic potentiation in the cerebral cortex and that combines repetitive electric nerve stimulation with transcranial magnetic stimulation (TMS). In contrast to the above mentioned studies, MS patients performed worse in clinical and paraclinical tests of motor function, but the enhancement of corticospinal excitability and the training-induced increments of motor performance were similar to controls. PAS-induced plasticity and motor learning did not correlate with motor impairment or CNS injury. Based upon their findings, the authors concluded that the early steps of neuronal plasticity are unlikely to limit the extent of compensatory changes in MS and therefore, rehabilitation efforts should focus on mechanisms supporting the later stages of motor learning (32).

An intriguing question of current research is whether rehabilitation procedures may induce and/or support compensatory adaptive changes. In this regard, evidence albeit limited is available that the clinical improvements of both, motor and cognitive rehabilitation, correlate with neural plasticity in the CNS of MS patients. Sastre-Garriga et al. investigated 15 MS patients and 5 healthy controls by functional magnetic resonance imaging (fMRI) with the PASAT paradigm. The cognitive rehabilitation program consisted of 15 computer-supported sessions and 5 non-computer-supported cognitive stimulation group sessions. After 5 weeks of cognitive training, patients showed significant clinical improvement of their neuropsychological performance, and this correlated to increased brain fMRI activity in several cerebellar areas (33). In a double-blind, randomized controlled trial of 12 MS patients, computer-assisted cognitive rehabilitation of attention deficits increased fMRI activity in the posterior cerebellum and in the superior parietal lobule in parallel to enhanced performance in attention abilities compared with 11 age- and gender-matched MS patients receiving a placebo intervention (34). Similarly, visuomotor performance improved after the first practice session of a visuomotor task (short-term practice) and after 2 weeks of daily sessions of the same task (longer-term practice) in both, 23 MS patients and 12 healthy controls. However, different relationships between the improvements of function and fMRI activity were found between the groups: in MS patients, increased function was associated with lower activation in the sensorimotor, posterior cingulate, and parahippocampal cortices, whereas in controls, greater long-term improvements correlated with smaller activation reductions in the visual cortex supporting the notion that even in MS patients with a high burden of pathology, brain plasticity is preserved, and that cognitive systems different from those of healthy controls contribute to this plasticity (35). However, despite the promising results that rehabilitation may indeed cause not only clinical improvement of cognitive and motor performance but also has distinct effects on brain activation, the role of fMRI in the context of clinical neurorehabilitation needs to be elucidated.

When summarizing the above mentioned findings, there is little doubt that plastic changes occur in the CNS, and that these changes may be modulated by practice. From a clinical point of view, it is obvious that patients undergoing neurorehabilitation improve with practice. Thus, these observations may bridge the gap between basic science and clinical experience. The results from basic studies may provide the scientific rationale to investigate recovery-oriented strategies in clinical trials and to implement them into rehabilitation measures. Several promising new rehabilitation techniques are examples of this approach: impairment-oriented training, CIMT, electromyogram-triggered neuromuscular stimulation, and robotic interactive therapies (2). It should be kept in mind that most of this evidence came from studies in patients with stroke or spinal cord injuries. However, more and more studies support the usefulness of these measures also in MS patients that reflect the clinical experience that we have made in our rehabilitation center during the last years (5). High-quality, carefully designed studies of the effectiveness of neurorehabilitation are necessary that should include both, clinical outcomes and neuroplastic measures. These studies may further move MS rehabilitation from empirical strategies toward evidence-based interventions and help to elucidate the basic mechanisms that are responsible for the clinical effects. Eventually, further research may provide the base to develop effective therapies that support the neuroplastic changes responsible for functioning, activity, and participation of persons with MS in order to reach and maintain their optimal physical, sensory, intellectual, psychological, and social functioning levels and promote the best possible quality of life.

## Conflict of Interest Statement

The author declares that the research was conducted in the absence of any commercial or financial relationships that could be construed as a potential conflict of interest.
